# Sex differences of vascular brain lesions in patients with atrial fibrillation

**DOI:** 10.1136/openhrt-2022-002033

**Published:** 2022-09-13

**Authors:** Selinda Ceylan, Stefanie Aeschbacher, Anna Altermatt, Tim Sinnecker, Nicolas Rodondi, Manuel Blum, Michael Coslovsky, Simone Evers-Dörpfeld, Sacha Niederberger, David Conen, Stefan Osswald, Michael Kühne, Marco Düring, Jens Wuerfel, Leo Bonati, SWISS-AF Investigators, Stefanie Aeschbacher

**Affiliations:** 1 Cardiology Division, Cardiovascular Research Institute Basel, University Hospital Basel, Basel, Switzerland; 2 Cardiovascular Research Institute Basel, University Hospital Basel, University of Basel, Basel, Switzerland; 3 MIAC AG and Department of Biomedical Engineering, University of Basel, Basel, Switzerland; 4 Institute of Primary Health Care (BIHAM), University of Bern, Bern, Switzerland; 5 Department of General Internal Medicine, Inselspital Universitatsspital Bern, Bern, Switzerland; 6 Cardiovascular Research Institute Basel, Department of Clinical Research, University of Basel, Basel, Switzerland; 7 Population Health Research Institute, McMaster University, Hamilton, Ontario, Canada; 8 Department of Neurology and Clinical Research, University of Basel, Basel, Switzerland; 9 Department of Neurology, Reha Rheinfelden, Rheinfelden, Switzerland

**Keywords:** Atrial Fibrillation, STROKE, Atrial Flutter

## Abstract

**Objective:**

To examine sex differences in prevalence, volume and distribution of vascular brain lesions on MRI among patients with atrial fibrillation (AF).

**Methods:**

In this cross-sectional analysis, we included 1743 patients with AF (27% women) from the multicentre Swiss Atrial Fibrillation study (SWISS-AF) with available baseline brain MRI. We compared presence and total volume of large non-cortical or cortical infarcts (LNCCIs), small non-cortical infarcts, microbleeds (MB) and white matter hyperintensities (WMH, Fazekas score ≥2 for moderate or severe degree) between men and women with multivariable logistic regression. We generated voxel-based probability maps to assess the anatomical distribution of lesions.

**Results:**

We found no strong evidence for an association of female sex with the prevalence of all ischaemic infarcts (LNCCI and SNCI combined; adjusted OR 0.86, 95% CI 0.67 to 1.09, p=0.22), MB (adjusted OR 0.91, 95% CI 0.68 to 1.21, p=0.52) and moderate or severe WMH (adjusted OR 1.15, 95% CI 0.90 to 1.48, p=0.27). However, total WMH volume was 17% larger among women than men (multivariable adjusted multiplicative effect 1.17, 95% CI 1.01 to 1.35; p=0.04). Lesion probability maps showed a right hemispheric preponderance of ischaemic infarcts in both men and women, while WMH were distributed symmetrically.

**Conclusion:**

Women had higher white matter disease burden than men, while volume and prevalence of other lesions did not differ. Our findings highlight the importance of controlling risk factors for cerebral small vessel disease in patients with AF, especially among women.

**WHAT IS ALREADY KNOWN ON THIS TOPIC**

- Clinically overt stroke, clinically unrecognised infarcts, microbleeds or other vascular brain lesions are commonly found on brain MRI in patients with atrial fibrillation (AF) and have a negative impact on cognitive function. The prevalence of covert brain lesions is increased in women in the general population.

**WHAT THIS STUDY ADDS**

- Our study provides data on differences in vascular brain lesions between men and women in a large sample of patients with AF, due to comprehensive quantity and quality of brain MRI at baseline. We observed a higher white matter disease burden in women than in men, despite well-anticoagulated study population. Volume and prevalence of other lesions showed no sex differences.

**HOW THIS STUDY MIGHT AFFECT RESEARCH, PRACTICE OR POLICY**

- Our findings emphasise the importance of targeting risk factors for cerebral small vessel disease among patients with AF, especially in women.

## Introduction

Atrial fibrillation (AF) is among the most common causes of overt ischaemic stroke worldwide and women with AF and more than one risk factor appear to be at greater risk of stroke than men.^1–3^ A recent study claims that female sex should be considered a risk modifier rather than an overall risk factor for stroke in patients with AF.^4^ Observations suggest that hormone therapy, menopause, haemodynamic and coagulatory mechanisms, cardiovascular remodelling, inflammation and less optimal treatment of vascular risk factors among female patients may contribute to a higher stroke risk in women with AF.^5^

In addition to clinically overt stroke, clinically unrecognised (ie, covert) infarcts, microbleeds (MB) or other vascular brain lesions are commonly found on brain MRI in patients with AF and have a negative impact on cognitive function.^6–8^ The prevalence of covert brain lesions is increased in women in the general population.^9^ However, data on differences in vascular brain lesions between men and women with AF are lacking. Such evidence may improve our understanding of sex-specific differences in vascular brain disease among patients with AF and help specifically target risk factors among women.

## Methods

### Study design and participants

This cross-sectional analysis used data of the Swiss Atrial Fibrillation cohort study (Swiss-AF), an ongoing prospective, observational cohort study in which patients with documented AF were enrolled between 2014 and 2017 across 14 centres in Switzerland. Detailed information on the study design has been published previously.^10^ In brief, 2165 patients ≥65 years and 250 patients aged 45–65 years with documented AF were recruited. Main exclusion criteria were short reversible forms of AF, acute illness within the last 4 weeks and inability to sign informed consent. Of 653 (27%) patients had to be excluded due to missing brain MRI at baseline. Nineteen patients were excluded because of other missing data, thus leaving 1743 patients for this analysis.

### Standard protocol approvals, registrations and patient consents

Patients or the public were not involved in the design, conduct, reporting or dissemination plans of our research.

### Clinical assessment

AF was classified as paroxysmal, persistent and permanent according to recommended definitions.^11^ Information about patient characteristics, comorbidities, lifestyle factors and current medications (antihypertensive medication, such as angiotensin-converting enzyme inhibitors, beta blockers, angiotensin—1-receptor blockers, calcium antagonists, diuretics, renin antagonists and aldosterone antagonists; oral anticoagulants including vitamin K antagonists and direct oral anticoagulants; statins and antiplatelet therapy) was acquired by standardised case report forms and validated questionnaires. Body height and weight were measured using standardised devices and body mass index was calculated as weight in kilograms divided by height in metres squared. Study physicians performed three consecutive blood pressure measurements at baseline, and the mean of them was used for this analysis.

### Brain MRI

MRI was done on 1.5 Tesla or 3.0 Tesla scanners. The standardised protocol consisted of a three-dimensional T1-weighted magnetisation-prepared rapid gradient echo (MPRAGE; spatial resolution 1.0×1.0×1.0 mm^3^), a two-dimensional axial fluid-damped inversion recovery (FLAIR; spatial resolution 1.0×1.0×3.0 mm^3^), and a two-dimensional axial diffusion-weighted imaging sequence (spatial resolution 1.0×1.0×3.0 mm^3^) with whole brain coverage and without interpolation. In addition, either a two-dimensional axial susceptibility-weighted imaging (spatial resolution 1.0×1.0×3.0 mm^3^) or a two-dimensional axial T2*-weighted gradient echo sequence (spatial resolution 1.0×1.0×3.0 mm^3^) was applied.

### Lesion segmentation

Scans were analysed centrally (MIAC AG; Medical Image Analysis Center AG, Basel, Switzerland). The blinded experts did not know any personal characteristics and standardised the lesions by marking and segmenting them according to an internal procedure approved for international clinical trials. Board-certified neuroradiologists confirmed all evaluations.^6 10^

Large non-cortical or cortical infarcts (LNCCIs) were defined as large non-cortical infarcts with a diameter >20 mm on axial sections as well as cortical infarcts characterised as hyperintense lesions on FLAIR involving the cortex regardless of their size and whether they also affect subcortical areas. Small non-cortical infarcts (SNCI) were defined as hyperintense lesions on FLAIR ≤20 mm in diameter on axial sections and not involving the cortex, compatible with ischaemic infarction in the area of a perforating arteriole (located in the white matter, subcortical grey matter or brainstem).^12^ Hyperintense lesions on FLAIR in either the periventricular or deep white matter region, brain stem or cerebellum, not meeting the criteria for LNCCIs or SNCIs, were identified as white matter hyperintensities (WMH) of presumed vascular origin,^12^ henceforth referred to as WMH. Perivascular spaces were differentiated from WMH by their tubular morphology, and subsequently excluded. WMH severity was classified using the Fazekas scale with a score of ≥2 and was defined as moderate to severe white matter disease.^6 12^ MBs were characterised as small (2 mm to 10 mm) areas of signal void with associated blooming seen on T2*-weighted MRI or other sequences that are sensitive to susceptibility effects (SWI-sequence).^13^ T2-weighted volumes of SNCIs, LNCCIs and WMH were segmented and quantified semiautomatically in mm^3^ using Amira (Mercury Computer Systems, Chelmsford, Massachusetts). Lesions with a central FLAIR hypointense core were segmented in total without differentiating between hyperintense and hypointense lesion areas. The normalised brain volume was estimated in cm^3^ on MPRAGE using SIENAX.^14^

### Image registration

FLAIR lesion masks of LNCCIs and SNCIs were registered linearly to a T_1_-weighted brain template using FSL (V.5.0, FMRIB’s Software Library; https://fsl.fmrib.ox.ac.uk/fsl/fslwiki). Then, the FLAIR-to-T1w images were non-linearly registered to an age-specific standard brain template. The same image processing steps were applied to the WMH masks. We did not map MBs due to their small size. Image registration was successful in 1716 (98.5%; 27% females) patients.

Voxel-based probability maps were computed by overlaying the normalised lesion masks of all patients—separately for infarcts (LNCCIs and SNCIs combined) and WMH. The percentage of lesions in the different vascular territories (calculated by the proportion of lesional voxels per vascular territory per patient) were computed for women and men.

### Statistical analysis

Baseline characteristics were compared between male and female patients. Depending on the distribution of the variables, continuous variables were presented as mean±SD or median (IQR). Categorical data were presented as numbers (percentage). Data were compared using χ^2^ test, t test or Mann-Whitney U test, as appropriate. Univariable and multivariable logistic and linear regression models were built to investigate the association of female sex with the prevalence and volume of brain lesions. Due to the skewed distribution, the volume of brain lesions was used as log-transformed variable and only analysed in patients with a prevalent ischaemic infarct or WMH. The OR (for the prevalence of brain lesions) and the β coefficient (for the log-transformed volume of brain lesions) were calculated with the corresponding 95% CIs, and female patients representing the reference group. Due to the log-transformed outcome variable (volume of brain lesions), β coefficients (95% CI) were reported as multiplicative effect (e ^∧^ β coefficient). Violin plots were built to show the distribution comparison of ischaemic lesions and WMH volume between female and male patients. P value was not adjusted for multiple testing. The regression analyses were adjusted for age, smoking status, body mass index, AF type, systolic blood pressure, history of hypertension, history of diabetes mellitus, history of sleeping apnoea, history of coronary heart disease, history of heart failure, oral anticoagulants, antiplatelet therapy, statin intake and antihypertensive treatment. Linear regression models using the volume of ischaemic lesions or WMH as the outcome variable were additionally adjusted for the total brain volume, to consider potential sex differences. For WMH volume, we tested an interacting effect of female sex and systolic blood pressure by including the respective interaction term in the model. As a sensitivity analysis, all analyses were repeated in patients without history of stroke or transient ischaemic attack (TIA).

Lesion probability maps were obtained for infarcts (LNCCI and SNCI combined) and WMH and compared between female and male patients. Patients with missing brain MRI or insufficient voxel analysis data have been excluded. For the voxel-based analyses, non-parametric and descriptive data like voxel percentage were reported as median and IQR. The Wilcoxon-matched pair rank test was used to compare median differences of voxel percentages within the vascular territory of anterior cerebral artery, middle cerebral artery, posterior cerebral artery, brainstem and cerebellum and to differ between the left and right hemispheres. Only patients with presence of any ischaemic infarcts and WMH were included in the analysis of the vascular territory distribution. Maximal overlay was calculated as maximum number of lesions per total of patients as a percentage occurred in a voxel. The numbers of the voxel-based mapping figures have been adjusted for the total remaining 1716 patients.

All analyses were performed on an available data basis and conducted using R V.4.0.3 (R Core Team, 2018, R Foundation, Vienna, Austria).

## Results

Baseline characteristics stratified by sex are presented in table 1. Mean age of the population was 73±8 years, 27.4% of participants were women and 90.1% were on oral anticoagulation (59.5% on new oral anticoagulants and 40.4% on Vitamin K antagonists). Compared with men, women were older (74 vs 72 years), had higher systolic blood pressure (138 mm Hg vs 134 mm Hg) and had more often paroxysmal AF (54.0 vs 42.7%). Men suffered more often from diabetes (17.5 vs 11.9%), coronary artery disease (32.0 vs 12.6%), sleep apnoea syndrome (15.4 vs 8.4%) and were more often treated with statins (52.3 vs 35.4%).

**Table 1:** Baseline characteristics stratified by sex

Variable	Women (n=478)	Men (n=1265)	P Value
Age (years)	74±8	72±9	<0.001
Body mass index (kg/m^2^)	26.7 (23.5, 31.2)	26.9 (24.6, 30.1)	0.35
Blood pressure systolic/diastolic (mm Hg)	138±21/78±12	134±18/79±12	<0.001/0.08
Smoking status, n (%)			<0.001
Current	39 (8.2)	92 (7.3)	
Past	163 (34.1)	680 (53.8)	
Never	276 (57.7)	491 (38.9)	
Average alcohol intake (drinks/day)	0.1 (0.0, 0.6)	0.7 (0.2, 1.6)	<0.001
Regular weekly physical activity, n (%)	229 (47.9)	617 (48.9)	0.77
Health Perception Score (0–100) *	70±17	74±17	<0.001
Atrial fibrillation type, n (%)			<0.001
Paroxysmal	258 (54.0)	540 (42.7)	
Persistent	140 (29.3)	406 (32.2)	
Permanent	80 (16.7)	319 (25.1)	
CHA_2_DS_2_-VASc score	4.0±1.5	3.1±1.7	<0.001
History of diabetes, n (%)	57 (11.9)	220 (17.5)	0.01
History of hypertension, n (%)	327 (68.4)	879 (69.5)	0.71
History of heart failure, n (%)	92 (19.3)	287 (22.7)	0.15
History of coronary artery disease, n (%)	60 (12.6)	405 (32.0)	<0.001
History of clinical stroke, n (%)	70 (14.6)	160 (12.6)	0.31
History of TIA, n (%)	50 (10.5)	110 (8.7)	0.27
History of major bleeding, n (%)	28 (5.9)	69 (5.5)	0.83
History of sleep apnoea, n (%)	40 (8.4)	195 (15.4)	<0.001
History of renal failure, n (%)	81 (17.0)	236 (18.7)	0.48
History/current hormonal therapy, n (%)	29 (6.1)	0 (0.0)	–
Cardiovascular medication, n (%)			
Antihypertensive medication	433 (90.6)	1100 (87.0)	0.05
Statins	169 (35.4)	662 (52.3)	<0.001
Antiarrhythmics class Ic and III	108 (22.6)	256 (20.2)	0.29
Oral anticoagulant intake, n (%)	432 (90.4)	1139 (90.0)	0.90
NOAC	276 (63.9)	659 (57.9)	0.04
Vitamin K antagonist	156 (36.1)	479 (42.1)	0.05
Antiplatelet (including aspirin), n (%)	57 (11.9)	250 (19.8)	<0.001

Values are mean ± SD, median (interquartile range) or n (%). The p value compares women and male patients. CHA_2_DS_2_-VASc score was defined as follows: female sex = 1 point; age ≥65 and <75 years = 1 point; age ≥75 years = 2 points, history of stroke or TIA = 2 points; history of heart failure = 1 point; hypertension = 1 point; diabetes = 1 point; vascular disease, consisting of history of myocardial infarction, history of percutaneous coronary intervention, history of coronary artery bypass graft surgery or periphery artery disease = 1 point.

*Health perception score is a self-assessment concerning their current state of health, evaluated in a scale from 0 to 100. Missing values: blood pressure systolic (n=11), diastolic (n=11), smoking (n=2), alcohol consumption (n=2), physical activity (n=2), CHA_2_DS_2_-VASc (n=2), heart failure (n=2), TIA (n=1), sleeping apnoea (n=1), renal failure (n=1). Antihypertensive medication includes angiotensin-converting-enzyme inhibitors, beta blockers, angiotensin-1-receptor-blockers, calcium antagonists, diuretics, renin antagonists, aldosterone antagonists.

NOAC, new oral anticoagulants; TIA, transient ischaemic attack.

**Figure 1:**
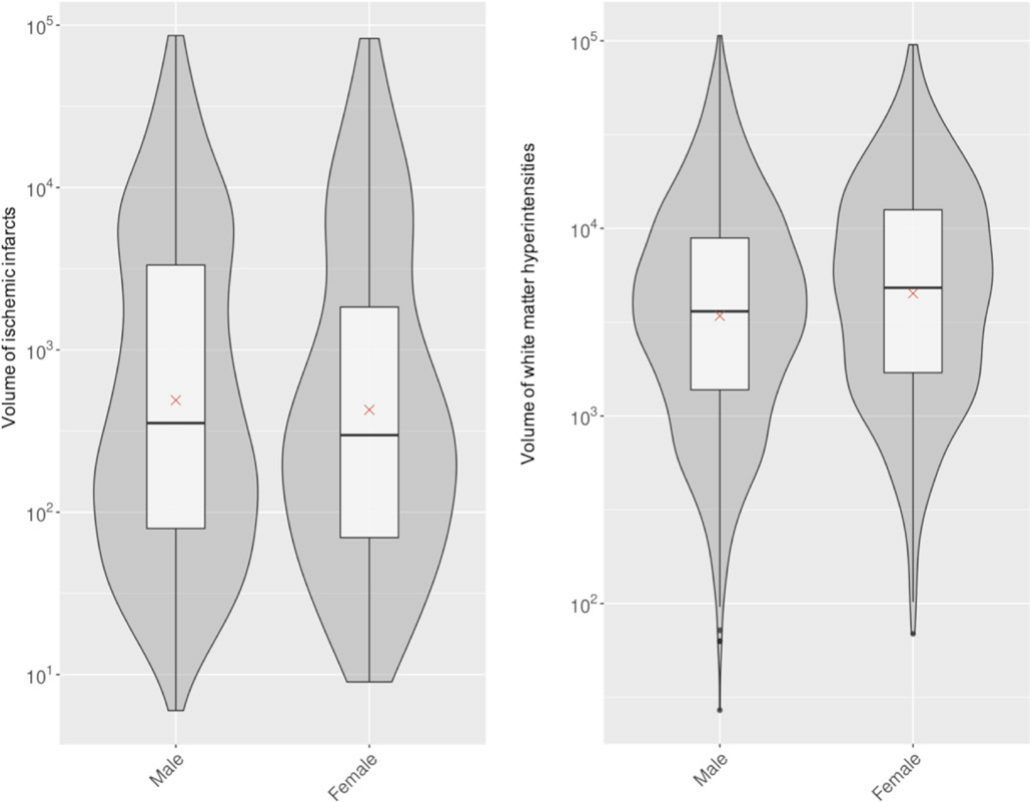
Violin plot of the log-transformed volume of ischaemic infarcts (LNCCI and SNCI combined) and WMH stratified by sex. The figure shows the distribution of ischaemic infarct volume (LNCCI and SNCI combined) and WMH volume in the SWISS-AF patients with successful co-registration (n=1716) compared between men and women. LNCCI, large non-cortical or cortical infarct; SNCI, small non-cortical infarct; SWISS-AF, Swiss Atrial Fibrillation study; WMH, white matter hyperintensity.

The prevalence and volume of vascular brain lesions are presented in table 2, as violin plots in figure 1 and unadjusted as well as adjusted comparisons thereof between women and men in table 3 and table 4. In the unadjusted analysis, we found no strong evidence for a difference between women and men in presence and volume of LNCCI (prevalence 20.3 vs 24.0%), SNCI (21.1 vs 22.8%), any ischaemic infarcts (LNCCI and SNCI combined, 35.6 vs 38.8%) or in prevalence of MB (21.0 vs 22.7%). Moderate or severe white matter disease was more common in women than in men (59.0 vs 51.7%, unadjusted p=0.006) and likewise, total WMH volume was greater in women (4779 mm^3^ vs 3609 mm^3^; multiplicative effect (1.32, 95% CI 1.2 to 1.5, unadjusted p<0.001).

**Table 2:** Prevalence and volume of brain lesions stratified by sex

Variable	Women (n=478)	Men (n=1265)
Large noncortical and cortical infarcts		
Prevalence, n (%)	97 (20.3)	303 (24.0)
Volume (mm^3^), median (IQR)	1230 (270, 7800)	1680 (258, 6971)
Small noncortical infarcts		
Prevalence, n (%)	101 (21.1)	289 (22.8)
Volume (mm^3^), median (IQR)	69 (30, 195)	69 (30, 189)
Any ischaemic infarcts (LNCCI or SNCI)		
Prevalence, n (%)	170 (35.6)	491 (38.8)
Volume (mm^3^), median (IQR)	299 (70, 1837)	354 (80, 3335)
Microbleeds		
Prevalence, n (%)	98 (21.0)	277 (22.7)
Counts (number)	1.0 (1.0, 2.0)	1.0 (1.0, 2.0)
White matter hyperintensities		
Prevalence, Fazekas scale ≥2, n (%)	282 (59.0)	653 (51.7)
Volume total (mm^3^), median (IQR)	4779 (1707, 12546)	3609 (1368, 8859)

Values are median (IQR) or n (%). Only the volume of patients showing the presence of lesions was taken into account. Missing values: microbleed count (n=55), white matter hyperintensities (n=1).

LNCCI, large noncortical and cortical infarcts (including acute lesions); SNCI, small noncortical infarcts (including acute lesions).

**Table 3:** Association between female sex and the prevalence of brain lesions

Prevalence	Univariable	Age adjusted model	Multivariable adjusted model
	All patients (n=1743) OR (95% CI)	All patients (n=1743) OR (95% CI)	All patients (n=1727) OR (95% CI)
Large noncortical and cortical infarcts	0.81 (0.62 to 1.04) p=0.11	0.73 (0.56 to 0.95) p=0.02	0.86 (0.65 to 1.14) p=0.28
Small noncortical infarcts	0.90 (0.70 to 1.17) p=0.44	0.78 (0.59 to 1.01) p=0.06	0.82 (0.62 to 1.09) p=0.18
Ischaemic lesions (LNCCI and SNCI)	0.87 (0.70 to 1.08) p=0.21	0.75 (0.60 to 0.94) p=0.01	0.86 (0.67 to 1.09) p=0.22
Microbleeds	0.91 (0.70 to 1.17) p=0.47	0.81 (0.62 to 1.06) p=0.13	0.91 (0.68 to 1.21) p=0.52
White matter hyperintensities, Fazekas≥2	1.35 (1.09 to 1.67) p=0.006	1.11 (0.88 to 1.40) p=0.37	1.15 (0.90 to 1.48) p=0.27

Data are presented as OR and 95% CI; predictor of interest: female sex; multivariable adjusted model was adjusted for age, body mass index, smoking status, AF type (paroxysmal vs non-paroxysmal), systolic blood pressure, hypertension, diabetes mellitus, heart failure, coronary heart disease, sleep apnoea, statin therapy, antihypertensive medication, oral anticoagulation, antiplatelet therapy. Missing values: microbleeds count (n=55); white matter hyperintensities (n=1); covariates (n=16).

AF, atrial fibrillation; LNCCI, large noncortical and cortical infarcts (including acute lesions); SNCI, small noncortical infarcts (including acute lesions).

**Table 4:** Association between female sex and the log-transformed volume of brain lesions

Volume	Univariable	Age adjusted model	Multivariable adjusted model
	Multiplicative effect (95% CI)	Multiplicative effect (95% CI)	Multiplicative effect (95% CI)
Large noncortical and cortical infarcts	0.96 (0.61 to 1.53) p=0.88	0.96 (0.60 to 1.53) p=0.86	1.13 (0.64 to 1.98), p=0.67
Small noncortical infarcts	0.98 (0.75 to 1.29) p=0.89	0.98 (0.75 to 1.29) p=0.89	1.20 (0.89 to 1.62), p=0.24
Ischaemic lesions (LNCCI and SNCI)	0.87 (0.59 to 1.29), p=0.50	0.88 (0.59 to 1.30), p=0.51	1.18 (0.76 to 1.85), p=0.46
White matter hyperintensities, total	1.32 (1.15 to 1.53) p<0.001	1.14 (1.01 to 1.30) p=0.04	1.17 (1.01 to 1.35), p=0.04

Data are presented as multiplicative effect and 95% CI; multiplicative effect=e^∧^ β-coefficient (due to log-transformed outcome variable); Only patients with the respective lesion were taken into account for this analysis; predictor of interest: female sex; multivariable adjusted model was adjusted for age, body mass index, smoking status, AF type (paroxysmal vs non-paroxysmal), systolic blood pressure, hypertension, diabetes mellitus, heart failure, coronary heart disease, sleep apnoea, statin therapy, antihypertensive medication, oral anticoagulation, antiplatelet therapy and normalised brain volume. Missing values multivariable adjusted models: LNCCI n=3; SNCI n=2; ischaemic lesions n=4; WMH n=16. Number of patients in the multivariable adjusted model (including brain volume): LNCCI: n=333; SNCI: n=350; ischaemic lesions: n=569; white matter lesions: n=1491.

AF, atrial fibrillation; LNCCI, large noncortical and cortical infarcts (including acute lesions); SNCI, small noncortical infarcts (including acute lesions).

In the age-adjusted comparison, the prevalence of LNCCI (OR 0.73, 95% CI 0.56 to 0.95, p=0.02) alone and of any infarct (LNCCI and SNCI combined, OR 0.75, 95% CI 0.60 to 0.94, p=0.01) was lower in women than in men, but the differences were no longer statistically significant after adjustment for additional patient characteristics (table 3). The association between female sex and presence of moderate or severe white matter disease was no longer significant after adjusting for age (OR 1.11, 95% CI 0.88 to 1.40, p=0.37) and after adjusting for additional patient characteristics (OR 1.15, 95% CI 0.90 to 1.48, p=0.27; table 3). However, women had greater total WMH volume than men both in the age-adjusted (multiplicative effect 1.14, 95% CI 1.01 to 1.30, p=0.04) and in the multivariable-adjusted model (multiplicative effect 1.17, 95% CI 1.01 to 1.35, p=0.04; table 4). There was no association between sex and volume of LNCCI, SNCI or any infarct. In a post hoc analysis, there was no interaction between female sex and systolic blood pressure and WMH volume as the outcome variable.

In the subgroup of patients without a history of stroke or TIA, the prevalence and volume of vascular brain lesions were lower than in the full study population, both among men and women (online supplemental table e-1). The associations between female sex and brain lesions were nearly similar to the full study population (online supplementary table e-2, e-3).

10.1136/openhrt-2022-002033.supp1 Supplementary data

**Figure 2:**
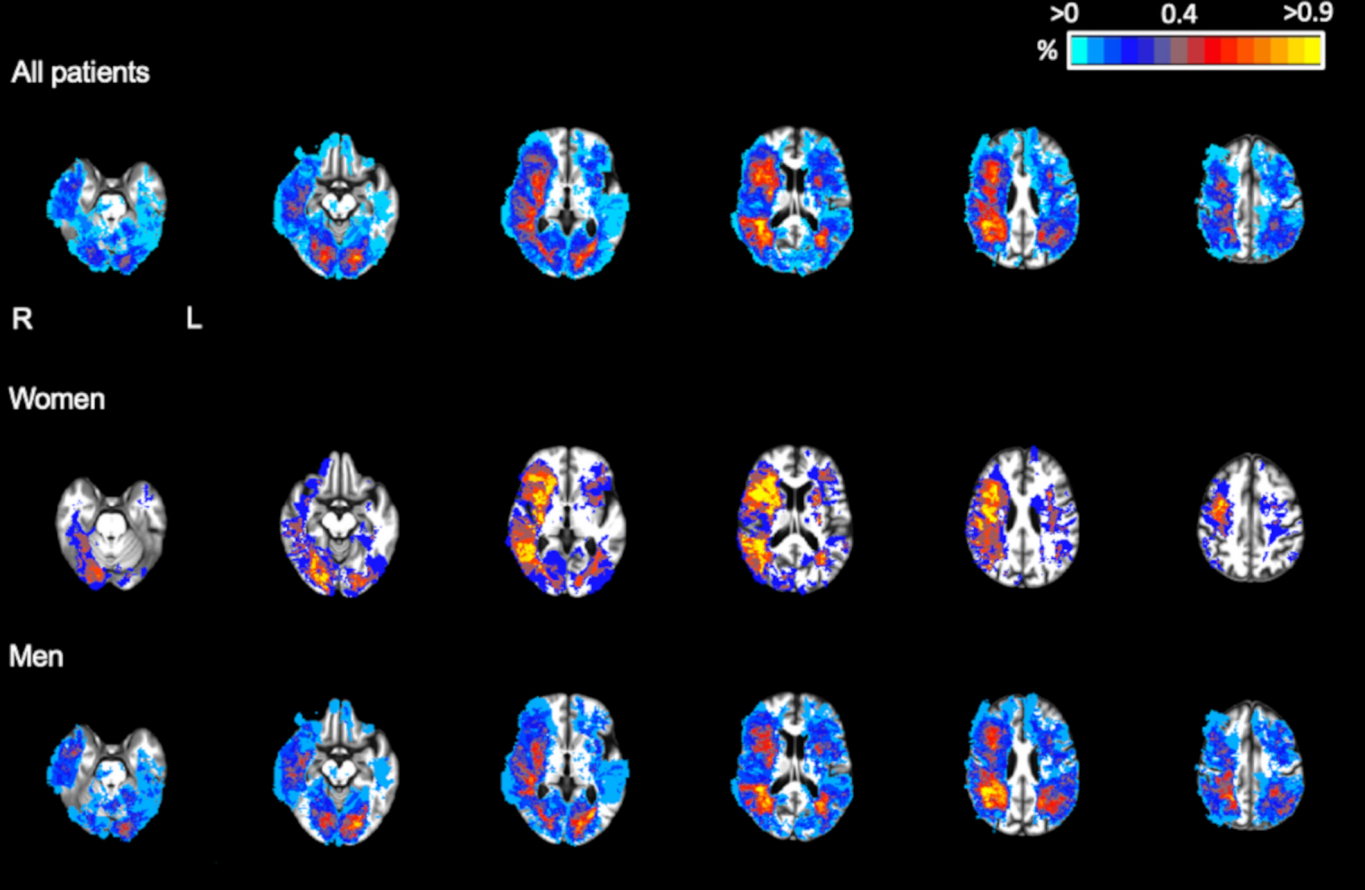
Localisation of ischaemic lesions (LNCCI and SNCI combined) stratified by sex. The figure shows the distribution of ischaemic lesions (LNCCI and SNCI, except acute lesions) in the SWISS-AF patients with successful co-registration (n=1716) compared between men and women in a standard space. The colour indicates that a voxel is affected by an ischaemic lesion in this percentage (%) of patients. LNCCI, large non-cortical or cortical infarct; SNCI, small non-cortical infarct; SWISS-AF, Swiss Atrial Fibrillation study.

Maximal achievable overlay of ischaemic lesions in a voxel was 1.3% overall (1.7% for women and 1.5% for men). Voxels affected by ischaemic lesions were similarly distributed in women and men, with a right-hemispheric predominance of infarcts in both sexes (figure 2). Total WMH maximal overlay was 65.3%, in women 76.4% and men 61.2%. An equal distribution for WMH was observed over the two hemispheres in men and women (online supplemental figure e-1).

10.1136/openhrt-2022-002033.supp2 Supplementary data

Percentages of voxels affected by infarcts and WMH within different vascular territories and brain regions by sex are listed in online supplemental table e-4 in the online supplement. There were no differences between men and women in the voxel-based prevalence of infarcts in any territory.

## Discussion

First, we did not find significant sex-related differences in the prevalence and volume of LNCCIs, SNCIs and MB. Second, women more often had moderate or severe white matter disease and larger volumes of WMH compared with men, the difference in the latter remaining significant after adjustment for baseline characteristics. Third, we did not observe any differences in regional distribution of vascular brain lesions between women and men, with infarcts predominantly occurring in the right hemisphere, and symmetrical location of white matter disease in both sexes.

Covert vascular brain lesions are present in a substantial proportion of patients with AF. We previously reported that 22% of patients in the Swiss-AF cohort study had LNCCIs and 21% SNCIs on MRI at study enrolment, despite the fact that most of the patients were taking oral anticoagulants (89%).^6^ Even after exclusion of patients with a history of stroke or TIA, these proportions were still 15% and 18%, respectively. Large-scale studies on sex-specific differences in vascular brain lesions among patients with AF have been lacking and the current evidence is largely limited to the risk of clinically overt stroke.

In the absence of other risk factors (ie, in the absence of non-sex related points of the CHA_2_DS_2_-VASc score), the risk of thromboembolic events in women with AF is low. However, female sex increases risk across the higher risk strata (especially in the presence of ≥2 non-sex-related stroke risk factors).^4^ In our study, the median CHA_2_DS_2_VASC score among women was 4±2% and 81% had ≥2 non-sex-related stroke risk factors. Nonetheless, the age-corrected prevalence of ischaemic infarcts was lower in women than in men. When correcting for additional patient characteristics and cardiovascular risk factors in the multivariable model, this difference was no longer significant. Hence, we found no sex differences of ischaemic brain infarcts on MRI. Both in women and men, the majority of these infarcts were covert, that is, present in patients without a history of stroke or TIA.

An important finding of our study was that women with AF had a higher burden of white matter disease than men. The median volume WMH was about a third larger in women. The Rotterdam Scan Study reported that women had a trend to a higher WMH burden than men in the general population.^15^ WMH by the definition used in our study are considered markers of cerebral small vessel disease.^12^ Age, hypertension and diabetes are among the most important risk factors for cerebral small vessel disease. In our cohort, women were older, less often diabetic but had higher baseline blood pressure than men. Nonetheless, in our study, the association between female sex and WMH volume persisted after correction for these as well as other risk factors and patient characteristics.

Although 91% of all women and 87% of all men in our cohort were on antihypertensive medication at baseline, systolic blood pressure at baseline was significantly higher in women than in men with a difference in median of 4 mm Hg. An important implication of our study is, therefore, that women with AF might be undertreated for hypertension. The Framingham study showed that women over the age of 60 have higher blood pressure than men, probably because menopause accelerates the onset of arterial stiffness, especially in those with history of hypertension.^16^ A randomised controlled trial showed that patients with a systolic blood pressure goal of <120 mm Hg had less increase in WMH volume over time compared with patients with a target of <140 mm Hg.^17^ The Rotterdam Scan Study observed in the general population that WMH and covert brain infarcts independently increase the risk of clinically manifest stroke in the future,^18^ reduce cognitive performance and are associated with brain volume loss.^19^ Our findings highlight that optimal care of patients with AF must go beyond anticoagulation and rhythm control and encompass vascular comorbidities causing vascular brain injury unrelated to embolism. Women appear to be vulnerable to cerebral small vessel disease, which might warrant lower targets for blood pressure control and other cardiovascular risk factors. Further studies may be needed to explore the influence of covert brain lesions and cardiovascular risk factors such as diabetes or smoking in women and men.

We found no difference in the regional distribution of vascular brain lesions between men and women. We found that in both sexes, ischaemic infarcts (LNCCI and SNCI) were more often located in the right hemisphere than in the left hemisphere. Physical properties of cardiac emboli, flow dynamics in the aortic arch, the more proximal origin of the brachiocephalic trunk compared with the left common carotid artery as well as the more rectilinear path of an embolus to the right compared with the left hemisphere may each contribute to this finding.^20 21^

Our study has limitations. The cross-sectional design precludes an assessment of causality or directionality of effect. Participants in our study were mostly white, and all were enrolled in Switzerland, limiting the generalisability of our findings to other populations. We only had data on prevalence of diabetes in our population, but no data on glycaemic control. The most important limitation was the low number of females participating in our cohort, a problem which is common to virtually all cardiovascular disease studies.^22^

In conclusion, we observed no strong differences between women and men with AF in the prevalence and volume of brain infarcts on MRI, the majority of which were clinically covert. However, women had a higher burden of white matter disease than men. Our findings emphasise the importance of targeting risk factors for cerebral small vessel disease among patients with AF, especially in women.

## Data Availability

Data are available upon reasonable request. Deidentified participant data are available from selinda.ceylan@hotmail.com. Swiss AF protocol is available in the supplementary materials.
