# Supplementary material for: Sex differences of vascular brain lesions in patients with atrial fibrillation
Source: Open Heart. 2022 Sep 13;9(2):e002033. doi: 10.1136/openhrt-2022-002033 (PMC9472202; doi:10.1136/openhrt-2022-002033)
Supplement: Supplementary data [file openhrt-2022-002033supp001.pdf]

Supplement

Sex differences of vascular brain lesions in patients with atrial fibrillation

Selinda Ceylan MD, Stefanie Aeschbacher PhD, Anna Altermatt PhD, Tim Sinnecker MD, Nicolas Rodondi MD MAS, Manuel Blum MD, Michael Coslovsky PhD, Simone Evers-Dörpfeld MD, Sacha Niederberger BMed, David Conen MD MPH, Stefan Osswald MD, Michael Kühne MD, Marco Düring MD, Jens Würfel MD, Leo H. Bonati MD, **for the SWISS-AF Investigators**

Table e-1: Prevalence and volume of brain lesions in patients without history of stroke/TIA

Variable	Women (n=370)	Men (n=1023)
**Large noncortical and cortical infarcts**		
Prevalence, n (%)	47 (12.7)	164 (16.0)
Volume (mm^3), median (IQR)	417 (180, 1189)	554 (155, 3933)
**Small noncortical infarcts**		
Prevalence, n (%)	62 (16.8)	199 (19.5)
Volume (mm^3), median (IQR)	66 (33, 167)	57 (30, 155)
**Any ischemic infarcts (LNCCI or SNCI)**		
Prevalence, n (%)	100 (27.0)	315 (30.8)
Volume (mm^3), median (IQR)	191 (56, 460)	162 (51, 814)
**Microbleeds**		
Prevalence, n (%)	66 (18.3)	205 (20.7)
Counts (Number), median (IQR)	1 (1, 2)	1 (1, 2)
**White matter hyperintensities**		
Prevalence, Fazekas scale ≥2, n (%)	204 (55.1)	494 (48.3)
Volume total (mm^3), median (IQR)	4287 (1603, 11263)	3309 (1242, 7956)

Values are median (interquartile range) or n (%). Only the volume of patients showing presence of lesions was taken into account. IQR = interquartile range; LNCCI = large noncortical and cortical infarcts (including acute lesions); SNCI = small noncortical infarcts (including acute lesions); TIA = transient ischemic attack. N=1393 patients without history of stroke or transient ischemic attack.

Table e-2: Association between female sex and the prevalence of brain lesions in patients without a history of stroke/TIA

	Univariable	Age adjusted model	Multivariable adjusted model
	**All patients (n=1393)**	**All patients (n=1393)**	**All patients (n=1379)**
**Prevalence**	**OR (95% CI)**	**OR (95% CI)**	**OR (95% CI)**
**Large noncortical and cortical infarcts**	0.76 (0.53; 1.07) p=0.13	0.69 (0.48; 0.98) p=0.04	0.81 (0.56; 1.18) p=0.29
**Small noncortical infarcts**	0.83 (0.61; 1.13) p=0.26	0.71 (0.51; 0.97) p=0.03	0.71 (0.50; 1.01) p=0.06
**Ischemic infarcts (LNCCI and SNCI)**	0.83 (0.64; 1.08) p=0.18	0.71 (0.54; 0.94) p=0.02	0.78 (0.58; 1.04) p=0.09
**Microbleeds**	0.86 (0.63; 1.17), p=0.34	0.77 (0.56; 1.05), p=0.10	0.86 (0.61; 1.20), p=0.38
**White matter hyperintensities (Fazekas ≥2)**	1.31 (1.03, 1.67), p=0.03	1.07 (0.82, 1.39), p=0.62	1.12 (0.85, 1.48), p=0.42

Data are presented as odds ratio and 95% confidence interval; Predictor of interest: Female sex; Multivariable adjusted model was adjusted for age, body mass index, smoking status, AF type (paroxysmal vs non-paroxysmal), systolic blood pressure, hypertension, diabetes mellitus, heart failure, coronary heart disease, sleep apnea, statin therapy, antihypertensive medication, oral anticoagulation, antiplatelet therapy. Missing values: microbleeds count (n=42); white matter hyperintensities (n=1); covariates (n=14). LNCCI = large noncortical and cortical infarcts (including acute lesions); SNCI = small noncortical infarcts (including acute lesions); CI = Confidence interval; OR = Odds Ratio; TIA = transient ischemic attack.

Table e-3: Association between female sex and the log-transformed volume of brain lesions in patients without a history of stroke/TIA

	Univariable	Age adjusted model	Multivariable adjusted model
**Volume (log – transformed)**	**Multiplicative effect (95% CI)**	**Multiplicative effect (95% CI)**	**Multiplicative effect (95% CI)**
**Large noncortical and cortical infarcts**	0.67 (0.37; 1.22) p=0.20	0.67 (0.37; 1.22) p=0.19	0.78 (0.37; 1.38), p=0.53
**Small noncortical infarcts**	1.12 (0.81; 1.54) p=0.49	1.12 (0.81; 1.55) p=0.48	1.24 (0.86; 1.79), p=0.25
**Ischemic lesions (LNCCI and SNCI)**	0.78 (0.51; 1.20) p=0.26	0.79 (0.51: 1.22) p=0.28	1.07 (0.65; 1.75), p=0.79
**White matter hyperintensities**	1.32 (1.12; 1.54), p<0.001	1.12 (0.97; 1.30), p=0.11	1.12 (0.95; 1.32), p=0.16

Data are presented as multiplicative effect and 95% confidence interval; multiplicative effect = e^ β-coefficient (due to log-transformed outcome variable); Only the volume of patients showing presence of lesions was taken into account; Predictor of interest: Female sex; Multivariable adjusted model was adjusted for age, body mass index, smoking status, AF type (paroxysmal vs non-paroxysmal), systolic blood pressure, hypertension, diabetes mellitus, heart failure, coronary heart disease, sleep apnea, statin therapy, antihypertensive medication, oral anticoagulation, antiplatelet therapy and normalized brain volume. Missing values multivariable adjusted models: covariates (n=3). LNCCI = large noncortical and cortical infarcts (including acute lesions); SNCI = small noncortical infarcts (including acute lesions); CI = Confidence interval; TIA = transient ischemic attack. Multivariable adjusted model (including brain volume): LNCCI: n=177 ; SNCI: n=239 ; Ischemic lesions: n=365 ; white matter hyperintensities: n= 1201.

Table e-4: Sex differentiated percentage of voxels affected by ischemic lesions and white matter lesions within different brain regions

**Ischemic lesions** (LNCCI and SNCI)	**Percentage %** Median (interquartile range)					
n = 588	*Women Left*	*Men Left*	*p-Value*	*Women Right*	*Men Right*	*p-Value*
**ACA**	0.02 (0.01; 0.14)	0.04 (0.01; 0.23)	0.09	0.02 (0.01; 0.07)	0.04 (0.01; 0.29)	0.36
**MCA**	0.03 (0.01; 0.24)	0.04 (0.01; 0.28)	0.18	0.07 (0.01; 0.44)	0.04 (0.01; 0.38)	0.39
**PCA**	0.13 (0.02; 0.50)	0.12 (0.02; 1.01)	0.19	0.07 (0.01; 1.40)	0.13 (0.01; 0.90)	0.56
**Brainstem**	1.34 (0.80; 1.88)	0.56 (0.08; 1.48)	0.49	0.09 (0.04; 0.10)	0.82 (0.45; 1.18)	0.90
**Cerebellum**	0.31 (0.09; 0.64)	0.23 (0.08; 0.64)	0.06	0.16 (0.11; 0.36)	0.24 (0.10; 0.77)	0.18

**WMH**	**Percentage %** Median (interquartile range)					
n = 1698	*Women Left*	*Men Left*	*p-Value*	*Women Right*	*Men Right*	*p-Value*
**ACA**	0.29 (0.07; 0.80)	0.18 (0.06; 0.55)	< 0.001	0.30 (0.10; 0.87)	0.23 (0.07; 0.62)	< 0.001
**MCA**	0.69 (0.23; 1.67)	0.45 (0.14; 1.12)	< 0.001	0.68 (0.27; 1.61)	0.48 (0.15; 1.13)	< 0.001
**PCA**	0.22 (0.10; 0.49)	0.22 (0.10; 0.45)	0.24	0.17 (0.07; 0.32)	0.18 (0.07; 0.33)	0.76
**Brainstem**	0.42 (0.24; 1.17)	0.48 (0.22; 0.88)	< 0.001	0.63 (0.30; 1.41)	0.37 (0.16; 0.98)	< 0.001
**Cerebellum**	0.09 (0.07; 0.14)	0.08 (0.05; 0.16)	0.53	0.06 (0.05; 0.22)	0.06 (0.04; 0.13)	0.97

Values are median percentage % (interquartile range). The p-value compares women and male patients. Only patients with presence of ischemic lesions were taken into account. Ischemic lesions n=588, Women n=154 (26%), Men n=434 (74%); WML n=1698, Women n=470 (28%), Men n=1228 (72%); LNCCI = large noncortical and cortical infarcts; SNCI = small noncortical infarcts; ACA = anterior cerebral artery; MCA = middle cerebral artery; PCA = posterior cerebral artery; WMH = white matter hyperintensities

Figure e-1: Localization of white matter hyperintensities stratified by sex

The figure shows the distribution of white matter hyperintensities in the SWISS-AF patients with successful co-registration (n=1716) compared between men (n=1245) and women (n=471) in a standard space. The color indicates that a voxel is affected by a white matter hyperintensity in this percentage (%) of patients.

Swiss-AF investigators

University Hospital Basel and Basel University: Stefanie Aeschbacher, Chloé Auberson, Steffen Blum, Leo H. Bonati, Selinda Ceylan, David Conen, Simone Evers-Doerpfeld, Ceylan Eken, Marc Girod, Elisa Hennings, Elena Herber, Vasco Iten, Philipp Krisai, Michael Kühne, Mirko Lischer, Christine Meyer-Zürn, Pascal Meyre, Andreas U. Monsch, Christian Müller, Stefan Osswald, Anne Springer, Christian Sticherling, Thomas Szucs, Gian Völlmin.
Principal Investigator: Stefan Osswald; Local Principal Investigator: Michael Kühne

University Hospital Bern: Faculty: Drahomir Aujesky, Manuel R. Blum, Urs Fischer, Juerg Fuhrer, Laurent Roten, Simon Jung, Heinrich Mattle; Research fellows: Luise Adam, Carole Elodie Aubert, Martin Feller, Axel Loewe, Elisavet Moutzouri, Claudio Schneider; Study nurses: Tanja Flückiger, Cindy Groen, Lukas Ehrsam, Sven Hellrigl, Alexandra Nuoffer, Damiana Rakovic, Nathalie Schwab, Rylana Wenger.
Local Principal Investigator: Nicolas Rodondi

Stadtspital Triemli Zurich: Christopher Beynon, Roger Dillier, Michèle Deubelbeiss, Franz Eberli, Christine Franzini, Isabel Juchli, Claudia Liedtke, Jacqueline Nadler, Thayze Obst, Jasmin Roth, Fiona Schlomowitsch, Xiaoye Schneider, Katrin Studerus, Noreen Tynan, Dominik Weishaupt. Local Principal Investigator: Andreas Müller

Kantonspital Baden: Simone Fontana, Silke Kuest, Karin Scheuch, Denise Hischier, Nicole Bonetti, Alexandra Grau, Jonas Villinger, Eva Laube, Philipp Baumgartner, Mark Filipovic, Marcel Frick, Giulia Montrasio, Stefanie Leuenberger, Franziska Rutz.
Local Principal Investigator: Jürg-Hans Beer

Cardiocentro Lugano: Angelo Auricchio, Adriana Anesini, Cristina Camporini, Giulio Conte, Maria Luce Caputo, Francois Regoli. Local Principal Investigator: Tiziano Moccetti

Kantonsspital St. Gallen: Roman Brenner, David Altmann, Michaela Gemperle. Local Principal Investigator: Peter Ammann

Hôpital Cantonal Fribourg: Mathieu Firmann, Sandrine Foucras, Martine Rime. Local Principal Investigator: Daniel Hayoz

Luzerner Kantonsspital: Benjamin Berte, Virgina Justi, Frauke Kellner-Weldon, Brigitta Mehmann, Sonja Meier, Myriam Roth, Andrea Ruckli-Kaeppeli, Ian Russi, Kai Schmidt, Mabelle Young, Melanie Zbinden. Local Principal Investigator: Richard Kobza

Ente Ospedaliero Cantonale Lugano: Jane Frangi-Kultalahti, Anica Pin, Luisa Vicari
Local Principal Investigator: Giorgio Moschovitis

University Hospital Geneva: Georg Ehret, Hervé Gallet, Elise Guillermet, Francois Lazeyras, Karl-Olof Lovblad, Patrick Perret, Philippe Tavel, Cheryl Teres. Local Principal Investigator: Dipen Shah

University Hospital Lausanne: Nathalie Lauriers, Marie Méan, Sandrine Salzmann. Local Principal Investigator: Jürg Schläpfer

Bürgerspital Solothurn: Andrea Grêt, Jan Novak, Sandra Vitelli. Local Principal Investigator: Frank-Peter Stephan

Ente Ospedaliero Cantonale Bellinzona: Jane Frangi-Kultalahti, Augusto Gallino. Local Principal Investigator: Marcello Di Valentino

University of Zurich/University Hospital Zurich: Fabienne Witassek, Matthias Schwenkglenks.

Medical Image Analysis Center AG Basel: Jens Würfel (Head), Anna Altermatt, Michael Amann, Marco Düring, Petra Huber, Esther Ruberte, Tim Sinnecker, Vanessa Zuber.

Clinical Trial Unit Basel: Michael Coslovsky (Head), Pascal Benkert, Gilles Dutilh, Milica Markovic, Pia Neuschwander, Patrick Simon

Schiller AG Baar: Ramun Schmid
