# Supplementary figures and images for: Sex differences of vascular brain lesions in patients with atrial fibrillation
Source: Open Heart. 2022 Sep 13;9(2):e002033. doi: 10.1136/openhrt-2022-002033 (PMC9472202; doi:10.1136/openhrt-2022-002033)

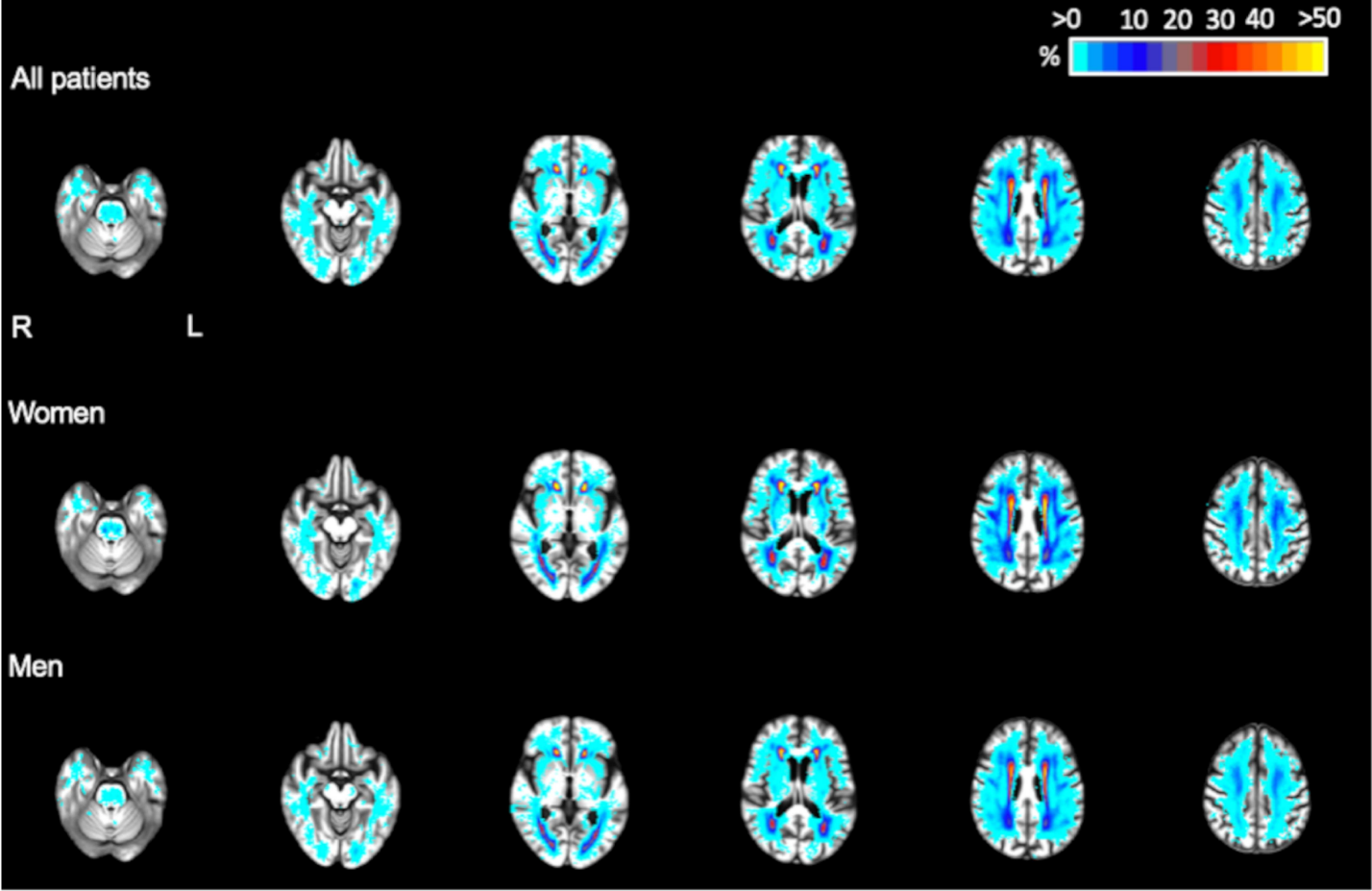

Supplement: Supplementary data [file openhrt-2022-002033supp002.pdf]
